# Clinical and Histopathological Features of Primary Oral Cancers in Shiraz, Iran: A 7- Years Retrospective Study

**DOI:** 10.31661/gmj.v13iSP1.3678

**Published:** 2024-12-30

**Authors:** Hossein Daneste, Ladan Jamshidi, Sara Maroufi, Azita Sadeghzadeh, Javad Moayedi

**Affiliations:** ^1^ Department of Oral and Maxillofacial Surgery, School of Dentistry, Shiraz University of Medical Sciences, Shiraz, Iran; ^2^ Oral and Dental Disease Research Center, Department of Oral and Maxillofacial Medicine, School of Dentistry, Shiraz University of Medical Sciences, Shiraz, Iran; ^3^ Central Research Laboratory, School of Medicine, Shiraz University of Medical Sciences, Shiraz, Iran

**Keywords:** Oral Cancer, Squamous Cell Carcinoma, Clinical Study, Histopathology

## Abstract

Background: This study aimed to assess the clinical and histopathological factors associated with primary oral cancer in Shiraz, southwest Iran. Materials and Methods: This retrospective cross-sectional observational study evaluated 100 patients with primary oral cancers at the Department of Oral and Maxillofacial Medicine of Shiraz University of Medical Sciences between January 2014 and December 2020. Data were collected from patient records, including age, sex, occupation, family history, spice use, smoking history, alcohol consumption, underlying diseases, lesion location, pathology grade, TNM stage, perineural invasion, time of diagnosis, number of surgeries, and history of radiotherapy and chemotherapy.Results: The average age of patients was 58.5±9.2 years (range 40-70 years), with 67% being men. Smoking, alcoholism, systemic diseases, spice use, and family history were present in 46%, 32%, 32%, 11%, and 11% of patients, respectively. Lymph node involvement was observed mostly in zone I (99%). The mandible was the most commonly affected site (46%), followed by the tongue (39%), buccal mucosa (23%), maxilla (20%), oral floor (18%), lower lip (7%), and upper lip (1%). Squamous cell carcinoma (SCC) accounted for 97% of cases, with other primary oral malignancies comprising the remaining 3%. Among the neoplasms, 55% were well-differentiated, while 45% were moderately differentiated.Conclusion: This study found that primary oral malignancies in Shiraz, Iran were predominantly found in men, with smoking and systemic diseases being major risk factors, and identified the mandible and tongue as the most common sites, with SCC being the most prevalent pathology, often well-differentiated.

## Introduction

Oral cancer is a leading cause of death among various malignancies, with smoking and alcohol consumption being the main risk factors. It is often undetected in its early stages, and delayed detection leads to complex treatment approaches that negatively impact patients’ survival rates [1][2]. Despite significant advances in diagnosis and treatment, oral cancers have a poor prognosis, with a five-year survival rate of 56% in the United States and Western Europe. Over the last 30 years, the incidence and prevalence of oral SCC have been increasing, particularly among younger individuals [3]. The most important prognostic factor is the level of tumor progression at diagnosis classified using the TNM system, which includes three basic clinical features: tumor size, lymph node metastasis, and distant metastasis. Other notable prognostic factors include tumor grade and the depth of tumor invasion [4][5].

Cytology smears are an additional method for diagnosing lesions, as morphological analysis increases the accuracy of evaluating cellular parameters such as nuclear size, cell diameter, nucleus and cytoplasm location, and nucleus-to-cytoplasm ratio [6]. SCC of the gingiva, mouth floor, buccal mucosa, and retromolar triangle can potentially spread to the mandible, invading the periosteum, foramen, attached mucosa, and cortical bone. Oral SCC typically follows a sequential metastatic pattern involving nodes I-V, with levels I-III posing the highest risk of metastasis. At the same time, nodules in the lower neck region have a lower risk of metastasis [7]. Perineural space involvement is a significant feature of invasive histopathology, indicating an increased risk of recurrence, postoperative complications, and mortality. Perineural involvement can be classified into clinical and subclinical categories based on the presence or absence of pain, hyperesthesia, dysesthesia, and motor nerve involvement [5]. The depth of invasion is a crucial histological feature that predicts tumor metastases. Thin oral SCCs with superficial invasion have a higher risk of regional lymph node metastasis compared to thick tumors that invade the underlying soft tissues. Survival analysis demonstrates that tumor thickness and stage are critical histopathological factors that describe the tumor and its prognosis. Tumors with a thickness of 2 mm or less have a 7.5% risk of latent nodal metastases, while those 3-8 mm thick carry a 27.5% risk, and a thickness of 9 mm or more leads to a 41.2% risk. These numbers emphasize the importance of determining tumor thickness [7]. As the current literature highlights the poor prognosis of oral cancers due to late detection and limited understanding of its clinical and histopathological features, particularly in specific geographic regions, we aimed to investigate the clinical and histopathological characteristics of primary oral cancers in Shiraz, Iran, a region with limited data on this topic.

## Materials and Methods

Ethics

The research protocol was approved by the local Ethics Committee of Shiraz University of Medical Sciences (approval no. IR.SUMS.DENTAL.REC.1399.167). It is worth noting that all patients involved in this study provided written informed consent based on the Declaration of Helsinki and its later amendments.

Patients

In this retrospective cross-sectional observational study, we studied all patients who were diagnosed with primary oral cancer by an oral and maxillofacial surgeon, oral medicine specialist, or pathologist between January 2014 and December 2020 in the Department of Oral and Maxillofacial Medicine of Shiraz University of medical sciences. Constitutive sampling was performed till a total of 100 patients met the inclusion criteria, which included a diagnosis of oral cavity malignancy, an age between 40 and 70 years, and primary tumor involvement. Patients without definitive confirmation of oral malignancy, individuals with other uncontrolled systemic diseases, or those with incomplete medical records were excluded from the study.

Data Collection

To collect data, we used a checklist with three sections based on the patient’s medical records. The first section included demographic and clinical information, such as age, sex, occupation, family history, and underlying diseases. To assess social habits related to oral cancer, we reviewed the patient’s medical records and conducted interviews to gather detailed information on smoking history, alcohol consumption, and the use of spices. The second part listed pathological findings, including lesion location and type, lymph node involvement, tumor grade, perineural invasion, TNM stage, and time of lesion diagnosis.

To evaluate specific zones of lymph node involvement, we reviewed the patient’s medical records and imaging studies. Lymph node involvement was categorized into four zones based on the American Joint Committee on Cancer (AJCC) and Union for International Cancer Control (UICC) TNM staging system:

*Zone I: Submental, submandibular, and upper jugular nodes.

*Zone II: Middle jugular nodes.

*Zone III: Lower jugular nodes.

*Zone IV: Supraclavicular nodes.

The final section documented treatment information such as surgery, radiotherapy, and chemotherapy. To assess the cause of death, we reviewed the medical records and documentation of each patient’s final status. Additionally, researchers followed up on the survival and recurrence of the disease by contacting the patients in the study and referring them to the hospital.

Statistical Analysis

All statistical analyses were conducted using the Statistical Package for the Social Sciences, version 22 (SPSS Inc., Chicago, USA). The data were analyzed using the chi-squared test, independent t-test, and one-way analysis of variance (ANOVA). The significance level was set at less than 0.05.

## Results

In this study, 100 patients were included with a mean age of 58 years (range 40-70 years). Of these patients, 67% were men and 33% were women. The mean age for men was 57 years, while for women it was 60 years, showing no significant age difference (P=0.170). The study revealed that 80% of patients had risk factors (social habits) for oral cancer. Specifically, 46% had a history of smoking, 32% had a history of alcohol consumption, 32% had systemic disease, 11% used spices, 11% had a family history, and 7% had other risk factors associated with oral cancer.

In terms of the site of involvement, the mandible was predominantly affected (46%), followed by the tongue (39%), buccal mucosa (23%), maxilla (20%), oral floor (18%), lower lip (7%), and upper lip (1%). Some patients had involvement at two or three sites simultaneously. Regarding lymph nodes, 99% had zone I involvement, 96% had zone II involvement, 76% had zone III involvement, and 12% had zone IV involvement. Nine patients experienced disease recurrence: five had SCC of the tongue and mouth floor, three had SCC of the tongue and mandible, and one had SCC of the maxilla.

The remaining surviving patients had no significant complications during follow-up.Seven men died, three of whom had SCC of the tongue (two with distant metastasis; one died due to chemotherapy and immunosuppression), two had SCC of the mandible and maxilla (they died due to general and systemic weakness, tumor recurrence, inability to eat, and treatment complications), and two had SCC of the maxilla only (one died due to distant metastasis to the pelvis and the other due to lymph node metastasis to the cervical lymph nodes and breathing problems). According to the results presented in Table-1, mandible and buccal mucosa sites were more involved in men, while women tended to maxillary and tongue cancers more frequently. Pathological findings indicated that 97% of patients had SCC, 2% had adenoid cystic carcinoma (ACC), and 1% had mucoepidermoid carcinoma (MEC).

The difference in the frequency distribution of pathological findings based on gender did not demonstrate a significant difference (P=0.68). Buccal mucosa tumors were more prevalent among men than women (P=0.02). Notably, 55% of the tumors were well-differentiated, while the remaining 45% were moderately differentiated. No grades of higher severity were observed. The prevalence of well-differentiated (69.7%) and moderately differentiated (52.2%) tumors was significantly higher among men (P=0.038). According to the TNM stage at diagnosis, 1% of patients were in stage I, 12% in stage II, 54% in stage III, and 33% in stage IV. Two patients underwent radiotherapy before surgery, while 92 patients did so after surgery. Five patients underwent chemotherapy before surgery, while 17 patients did so after surgery, and 78 patients did not receive chemotherapy at all. At least a month had passed since the diagnosis of the disease in all patients.

## Discussion

**Table:** Table1. The Prevalence of Oral Cancers between Men and Women

**Parameters**		**Total (n=100)**	**Men (n=67), n(%)**	**Women (n=33) , n(%)**	**P-value**
	Maxilla	20(20%)	9(13.43%)	11(33.33%)	0.2
	Mandible	46(46%)	35(52.24%)	11(33.33%)	0.074
	Upper Lip	1(1%)	1(1.49%)	0(0%)	0.48
**Involvement Site**	Lower Lip	7(7%)	3(4.48%)	4(12.12%)	0.56
	Tongue	39(39%)	14(20.9%)	25(75.76%)	0.62
	Oral Floor	18(18%)	11(16.42%)	7(21.21%)	0.05
	Buccal Mucosa	23(23%)	20(29.85%)	3(9.09%)	0.02
	SCC	97(97%)	65(97.01%)	32(96.97%)	0.68
**Pathological Findings**	ACC	2(2%)	1(1.49%)	1(3.03%)	
	MEC	1(1%)	1(1.49%)	0(0%)	
**Pathology Grade**	Well	55(55%)	32(47.76%)	23(69.7%)	0.038
	Moderate	45(45%)	35(52.24%)	10(30.3%)	
	Stage 1	1(1%)	0(0%)	1(3.03%)	0.47
**TNM Stage**	Stage 2	12(12%)	8(11.94%)	4(12.12%)	
	Stage 3	54(54%)	38(56.72%)	16(48.48%)	
	Stage 4	33(33%)	21(31.34%)	12(36.36%)	

Squamous Cell Carcinoma **(SCC)**, Adenoid Cystic Carcinoma **(ACC)**, Mucoepidermoid Carcinoma **(MEC)**

Oral cancer is the most common form of head and neck cancer, typically associated with old age. Various factors contribute to the age at which these cancers develop, including diet, alcohol and tobacco use, and cultural circumstances within the community [8].

In the current study, the average age of patients was 58 years, with no statistically significant difference between men (57 years) and women (60 years). Consistent with our findings, several studies have reported that the mean age of women with oral SCC is higher than that of men [9][10][11][12].

The distribution of oral SCC varies across different regions of the world and appears to be more common in men than in women [8]. In 1950, the ratio of oral cancer in men to women was 6 to 1, but recent studies have shown that this ratio has dropped to less than 2 to 1 [10][13][14].

In our study, the men-to-women ratio was 2 to 1. However, this ratio was 5:1 in the Fars province of Iran [15], and 1.5 to 1 in Sari [16]. In a study conducted by Jacobson et al. [17], the men-to-women ratio was 2.5 to 3, while in Spain, it was an 8.4 to 1 ratio of men to women [18]. The present study revealed that 46% of primary oral cancer patients had a history of smoking, 32% reported alcohol consumption, and 40% had both habits. This underscores the important role of alcohol consumption, smoking, and their combined effects in the development of oral SCC. Consistent with our findings, Shiva et al. [16] found that out of six smokers, five had SCC and the remaining individual had salivary adenocarcinoma.

Other large-scale studies have also demonstrated a higher prevalence of smoking among patients with oral cancer [19][20][10][21][22]. A study at the University of Maryland involving 200 cases of oral SCC found that 161 patients were smokers, with many also reporting a history of alcohol consumption [23].

Most cases in our study had mandibular involvement (46%), followed by the tongue (39%), buccal mucosa (23%), maxilla (20%), and oral floor (18%). Shenoi et al. [24] also found that the mandibular bone was the most common site of oral cancer in India. Razavi et al. [25] identified the alveolar mucosa as the predominant site, followed by the tongue, and buccal mucosa. Sargaran et al. [11] reported that the alveolar ridge and vestibule were the most common sites, followed by the tongue. Other studies from Iran [26][27][19], the Netherlands [28], the Basque [18], the United Kingdom [29], and Sri Lanka [30] also indicated that oral SCC most frequently affects the tongue. However, studies from Thailand [31], Taiwan [32], and India [33] showed that the buccal mucosa was the most common site. This variation may be due to different geographical characteristics and oral habits such as chewing tobacco.

It seems that the tongue is one of the most affected sites, with differences between smokers and non-smokers. Specifically, the tongue and mouth floor are the most common sites in smokers and non-smokers, respectively. Additionally, the tongue and alveolar mucosa are the predominant sites in young and old patients, respectively [34].

According to our study, 99% of patients had involvement in Zone I, 96% in Zone II, 76% in Zone III, and 12% in Zone IV lymph nodes. Paying attention to the specific lymphatic chain involved can help diagnose the primary lesion. For instance, when considering zone IV involvement, one should consider nasopharyngeal lesions in the posterior cervical region, tonsil lesions, and anterior neck lymph nodes [35]. Additionally, the location of the metastatic mass is crucial for identifying the primary tumor [35][36][37].

In a study conducted by Ghazizadeh et al. [38], the most common site of a neck mass in both genders was the jugulodigastric lymphatic chain, with a prevalence of 20.37% in men and 21.98% in women. Another study found that 69% of lymph nodes extended into the extracapsular region, with no significant relationship between the size of cervical lymph nodes and the location of primary tumors in the tongue or lip [2].

Some studies suggest a high incidence of malignancy in posterior triangular neck masses [38]. Supraclavicular masses, which may be observed in young individuals and children, should be closely monitored [39][40]. It is recommended that if these masses are singular, asymmetrical, and growing rapidly, immediate surgical evaluation is warranted.

In our study, 97% of the pathological findings were SCC.

Previous studies have reported varying prevalence rates of SCC in different countries: 43% in Nigeria [9], 58% in Congo [41], 70% to 77% in Iran [42][11][43][25], 93% in the United States [44], and 98% in Australia [45]. These differences could be attributed to variations in sampling methods and study populations.

It is important to note that our study was conducted at a single medical center in Shiraz, Iran, which may limit the generalizability of our findings. In our study, 1% of cases were detected in stage I, 12% in stage II, 54% in stage III, and 33% in stage IV. Another study conducted in Shiraz found that most patients in both age groups (below and over 45 years) were diagnosed with stage IV [46].

Consistent with our findings, several studies have reported that the majority of patients are diagnosed in stages III and IV of the disease [47][1][31][48][49], underscoring the need for more extensive treatments.

It appears that lesions in the mouth often go unnoticed by both patients and dentists during routine examinations, leading to delayed diagnosis upon admission. Based on the studies reviewed, it appears that buccal mucosa tumors are more common in males than females (50, 51), as well as our findings. One limitation of the present study was the inability to assess certain risk factors due to its retrospective nature.

Another limitation was conducting the study in only one center; the patients in this center may not be representative of all patients with primary oral cancer in Shiraz. Therefore, future studies should be conducted in multiple centers and cities.

Due to variations in disease profiles in different regions, gaining a detailed understanding of the clinicopathological features of oral malignancies is essential for better evaluation and management.

## Conclusions

This study revealed that the majority of patients with primary oral malignancies were men. Smoking and systemic diseases were identified as the main risk factors. The mandible was the most frequently affected site, followed by the tongue, buccal mucosa, maxilla, and oral floor.

SCC was the most prevalent pathology, found in 97% of cases, with most cases being well-differentiated. Gender was shown to have a significant impact on both pathology and grade.

## Acknowledgment

The authors would like to thank the Vice-Chancellor of Research at Shiraz University of Medical Sciences, Shiraz, Iran for their financial support.

## Conflict of Interest

None of the authors have any conflicts of interest to disclose.
